# Navigating the challenges of clinical trial professionals in the healthcare sector

**DOI:** 10.3389/fmed.2024.1400585

**Published:** 2024-06-03

**Authors:** Galo Peralta, Blanca Sánchez-Santiago

**Affiliations:** ^1^Central Support Unit, Instituto de Investigación Marqués de Valdecilla (IDIVAL), Santander, Spain; ^2^Clinical Pharmacology Service, Clinical Trials Unit, Hospital Universitario Marqués de Valdecilla, Instituto de Investigación Marqués de Valdecilla (IDIVAL), Santander, Spain

**Keywords:** clinical trial, investigator, clinical research coordinator, clinical trial monitoring, nurse, pharmacist, ICH (international conference of harmonization) guidelines

## Abstract

Clinical trials (CTs) are essential for medical advancements but face significant challenges, particularly in professional training and role clarity. Principal investigators, clinical research coordinators (CRCs), nurses, clinical trial pharmacists, and monitors are key players. Each faces unique challenges, such as maintaining protocol compliance, managing investigational products, and ensuring data integrity. Clinical trials’ complexity and evolving nature demand specialized and ongoing training for these professionals. Addressing these challenges requires clear role delineation, continuous professional development, and supportive workplace environments to improve retention and trial outcomes. Enhanced training programs and a collaborative approach are essential for the successful conduct of clinical trials and the advancement of medical research.

## 1 Introduction

CTs are typically conducted within a healthcare setting where the researchers are usually clinicians, being the healthcare system responsible for two missions: improving patients’ health and advancing therapeutics, often in collaboration with the industry. This dual purpose of clinical assistance and research requires adequate support ecosystems characterized by a highly demanding organizational framework (1, 2) that has as a benchmark the Guideline for Good Clinical Practice (GCP) of the International Council for Harmonization of Technical Requirements for Pharmaceuticals for Human Use for Conducting Clinical Trials Guidelines (ICH) (3).

GCP is the international standard for ethical and scientific quality in clinical trial design, conduct, and reporting to protect trial participants’ rights, safety, and well-being (4). On the other hand, different regulations on CTs, such as Title 21, Chapter 1 of the Code of Federal Regulation (5) in the United States, and Regulation (EU) No 536/2014 of the European Parliament and of the Council of 16 April 2014 on clinical trials on medicinal products for human use (6) in Europe frame the definition of the responsibilities of the different participants in CTs. These regulations emphasize the necessity for clinical trials to be conducted by professionals who are not only ethically and scientifically trained but also well-versed in the nuances of clinical trial execution.

In this sense, the Declaration of Helsinki highlights the need for adequately trained professionals. It explicitly indicates that “*Medical research involving human subjects must be conducted only by individuals with the appropriate ethics and scientific education, training, and qualifications. Research on patients or healthy volunteers requires the supervision of a competent and appropriately qualified physician or other health care professional.*”(7).

As regulation, complexity, and requirements in CTs are increasing, support structures and professional expertise are more demanding (8–10), especially in the deployment of initial phases of clinical research (11). Moreover, new challenges such as centering opportunities around people and equity, guaranteeing accountability, and improving efficiency must be addressed (12). In this scenario, specific professional profiles are recommended to provide adequate support for CTs, such as principal investigators, sub-investigators, clinical study nurses, clinical research coordinators, study coordinators, pharmacists, and clinical monitors (clinical research associates) (13–16).

However, the literature recognizes that there are no standard definitions of the different profiles involved in CTs, highlights an overlap of functions, and points out the lack of specific training for these professionals involved in the development of CTs and a standard organizational model for defining high-quality support teams (2, 13, 17–19). Despite the relevance of the professional profiles and roles in CTs, there still needs to be literature reviews that address regulations, activities, profiles, qualifications required, training opportunities, and challenges in this field.

As we noted that the aggregate information available is quite limited, we consulted with Chat GPT-4, now recognized as a source of information for healthcare professionals (20). We asked Chat GPT-4: “Which are the main CT professional support profiles involved in implementing CTs in the clinical arena?” (21). The support profiles identified by Chat GPT-4 are reflected in Table 1. During a conversation with Chat GPT-4, it came to our attention that not all of the professionals identified are necessarily directly involved in the clinical setting. So then, we asked Chat GPT-4 to provide us with the outstanding profiles of the referred in Table 1. The second question launched to Chat GPT-4 was: Which of the profiles listed are the more relevant in the clinical arena? The top five profiles selected by Chat GPT-4 were: principal investigator, CRC, clinical research associate, biostatistician, and regulatory affairs specialist. This result showed discrepancies with the information we had previously gathered from the literature review, underscoring the importance of reviewing multiple sources to understand this topic comprehensively.

**TABLE 1:** Professional clinical trial support profiles in the clinical arena identified by Chat GPT-4.

Principal investigator
Clinical research coordinator or study coordinator
Nurses and clinical staff
Clinical research associate
Pharmacovigilance and safety officer
Medical monitor
Data manager
Biostatistician
Quality assurance professional
Regulatory affairs specialist
IT and Informatics specialists

This paper aims to thoroughly explore the intricate roles and responsibilities that define the backbone of clinical trial operations. It discusses the distinct challenges these professionals face, from ensuring rigorous protocol compliance and managing sensitive investigational products to safeguarding participant well-being and data accuracy. Additionally, it highlights the critical need for ongoing professional development and robust institutional support to sustain workforce competence and morale amidst the pressures of clinical trial demands.

## 2 Methods

For the initial phase of the review, PubMed was utilized to identify relevant published literature between 2000 and 2023. The search strategy involved specific descriptors related to each of the professional profiles being analyzed (“investigator”, “Clinical Research Nurse”, Clinical Trial Nurse”, Clinical Research Coordinator”, “Clinical Trial Pharmacist”, “Clinical Trial Monitor”, “Clinical Research Associates”) with the following descriptors: “qualifications,” “profiles,” “roles,” “challenges,” and “training.” This approach ensured a focused retrieval of relevant literature.

Additionally, the search included an examination of the existing literature reviews on the different CT professional profiles and a thorough analysis of the bibliographies of the identified documents to capture further pertinent sources. This iterative process of reviewing bibliographies helped to identify additional relevant materials that might not have been captured in the initial database searches.

Moreover, the review was extended to examine major international regulations pertinent to the professional profiles under discussion, specifically the guidelines from the International Council for Harmonization (ICH) and key regulations from European and US authorities. This aspect of the methodology ensured that the review was informed by current regulatory frameworks, which could impact professional roles and responsibilities.

Original documents related to the topics being analyzed were also scrutinized to ensure the inclusion of primary sources and firsthand information, which provided a solid foundation for the analysis and conclusions of the review. This comprehensive methodological approach facilitated a thorough exploration of the professional profiles, highlighting various challenges and training needs within the context of current regulatory and professional standards.

## 3 Principal investigator

### 3.1 Principal investigator role

GCP (3), the Code of Federal Regulations in the United States (22), Regulation (EU) No 536/2014 of the European Parliament (6), and other international standards, guidelines, and regulations (23–28), establish that the principal investigator’s responsibilities in CTs are broad. They include adequate expertise, providing resources needed, deploying the trial tasks in compliance with the protocol accordingly regulation and reporting, and guaranteeing high standards of medical care (Table 2).

**TABLE 2:** Principal investigator responsibilities in clinical trials [adapted from (3)].

Qualifications and Agreements: adequate qualification to assume responsibility, thoroughly familiar with the appropriate use of the investigational product(s), aware of GCP and regulation, permit monitoring and inspections and maintain list of persons delegated
Adequate Resources: recruiting capabilities, available time and qualified staff for the CT, informed team, supervise delegated trial-related duties
Medical Care of Trial Subjects: responsible for medical decisions and adequate adverse events management
Communication with the Institutional Review Board (IRB): written approval from the IRB that must be informed of the CT documents
Compliance with Protocol: sign the protocol and conduct the trial in compliance with the protocol without deviation without agreement by the sponsor and adequate explanations and documents
Investigational Product(s): responsibility for investigational product accountability at the trial site (records delivery, inventory, storing, use and return unused) that may be delegated to a pharmacist.
Randomization Procedures and Unblinding: the investigator should follow the trial’s randomization procedures
Informed Consent of Trial Subjects: comply with the applicable regulatory requirements and should adhere to GCP and to the ethical principles. Stay vigilant to avoid unduly influence a subject to participate, use a non-technical understandable language. Obtain written informed consent prior to a subject’s participation.
Records and Reports: maintain adequate and accurate source documents and trial records, ensuring the accuracy, completeness, legibility, and timeliness of data, tracing changes of Case Report Form.
Progress Reports: submit written summaries of the trial status to the IRB periodically and when the IRB require them.
Safety Reporting: report immediately all serious adverse events to the sponsor
Premature Termination or Suspension of a Trial: inform the institution and IRB promptly to the termination
Final Report(s) by Investigator: inform the institution, the IRB and regulatory authorities with a summary of the trial’s outcome.

In the US, the investigator must sign Form FDA 1572, an agreement to provide certain information to the sponsor and to assure that he/she will comply with FDA regulations related to the conduct of a clinical investigation of an investigational drug or biologic (22, 25, 29) (Table 3). The FDA 1572 has two purposes: (1) to provide the sponsor with information about the investigator’s qualifications and the clinical site that will enable the sponsor to establish and document that the investigator is qualified and the site is an appropriate location where to conduct the clinical investigation, and (2) to inform the investigator of his obligations and obtain the investigator’s commitment to follow pertinent FDA regulations (29).

**TABLE 3:** Commitments assumed by the investigator in the FDA 1572 form (29).

I agree to conduct the studies in accordance with the relevant, current protocol(s) and will only make changes in a protocol after notifying the sponsor, except when necessary to protect the safety, rights, or welfare of subjects.
I agree to personally conduct or supervise the described investigation(s).
I agree to inform any patients, or any persons used as controls, that the drugs are being used for investigational purposes and I will ensure that the requirements relating to obtaining informed consent and IRB review and approval are met.
I agree to report to the sponsor adverse experiences that occur during the investigation in accordance with regulation. I have read and understand the information in the investigator’s brochure, including the potential risks and side effects of the drug.
I agree to ensure that all associates, colleagues, and employees assisting in the conduct of the studies are informed about their obligations in meeting the above commitments.
I agree to maintain adequate and accurate records by regulation and to make those records available for inspection.
I will ensure that an IRB that complies with the requirements of regulation will be responsible for the initial and continuing review and approval of the clinical investigation. I also agree to promptly report to the IRB all changes in the research activity and all unanticipated problems involving risks to human subjects or others. Additionally, I will not make any changes in the research without IRB approval, except where necessary to eliminate apparent immediate hazards to human subjects.
I agree to comply with all other requirements regarding the obligations of clinical investigators and all other pertinent requirements

Accordingly to European (6) and US (5) regulations, the sponsor is responsible for selecting an investigator for each CT, who may further appoint one or more sub-investigators at the trial site to perform crucial trial-related procedures and make critical decisions. These sub-investigators could be associates, residents, or research fellows. However, it is of utmost importance that these tasks are performed in a manner that prioritizes the safety of the subjects and the reliability and robustness of the data generated at the CT site (3, 6, 30).

The CT team is under the investigator’s responsibility. In this sense, the Food and Drug Administration (FDA) (28) and the GCP (3) emphasize this role in the designation and supervision, ensuring that any individual delegated is qualified by education, training, and experience. The CT team is responsible for managing the daily activities of the trial; however, the investigator is ultimately responsible for ensuring that the trial is conducted in compliance with European (6) and US (5) regulations and that accurate records are maintained. The investigator is responsible for protocol violations or discrepancies (25). The FDA specifically focuses on four areas of investigator’s supervision: adequate delegation of study-related tasks, adequate qualification and training of the CT staff to conduct and understand the study, adequate supervision and involvement in the ongoing study, and adequate supervision or oversight of any third parties involved (28).

Informed consent is one of the critical responsibilities of the investigator in CTs (3, 30). Conducting research is an important endeavor, but it is crucial to prioritize the well-being of the participants and to ensure the purpose and risks of the study are disclosed and discussed with them. International regulation highlights that the investigator must inform through informed consent about the purpose of the research, the expected duration of the subject’s participation, the procedures to be followed, and any foreseeable risks or discomforts to the subject (5, 6, 31). Furthermore, it is the investigator’s responsibility to make it clear that patients’ decision to participate will not affect their future medical care. It is indispensable to avoid any hint of coercion when presenting the study, as patients often trust their doctors and may feel compelled to participate if asked (32).

The literature suggests that informed consent forms are often unclear and poorly understood by participants, emphasizing the need for improving communication and comprehension (33). Even more, some subjects may not even be aware that they are participating in research, highlighting the ethical imperative for investigators to ensure transparency and understanding (34). The investigator may delegate informed consent to team members adequately qualified by education, training, and experience. If present, information related to the investigator’s financial relationships or interests must be provided to the participants through informed consent (35). The roles of investigators in the informed consent process are particularly important in some areas, such as pediatric oncology, where doctors must balance their clinical and research roles, highlighting the need for transparent communication and ethical behavior (36). Also, the context of emergency CTs has been challenging for investigators (37).

Reporting is also a relevant responsibility of the investigator in CTs (3, 25). It includes reporting of adverse events by the investigator to the sponsor and the IRB (38), which is required by the different regulatory agencies (5, 6). These reports of severe events must occur without undue delay as they can affect the safety of the volunteers involved in the CTs and even the trial’s pertinence (39). Other reports that are the investigator’s responsibility are progress reports (usually annually) and the final report to inform the Institution and the IRB (3, 5, 38). Financial disclosures to the sponsor are also relevant and should be updated during the investigation and after the completion of the study according to different regulations (25).

### 3.2 Principal investigator qualification and training

The FDA (5), the European regulation (6), and GCP (3) require investigators qualified by training, education, and experience, although these normative and recommendations do not detail the competencies requirements. Moreover, the qualification standards for investigators vary widely among countries (40, 41). Generally, European countries demand GPC training every 2–3 years (41), and in the US, the National Center for Advancing Translational Science (NCATS) recommends undergoing training every 3 years (14). The IRB is responsible for reviewing the principal investigator’s qualifications as part of the trial application to the IRB and can ask questions and request documents such as curriculum vitae (3, 35).

The investigator is usually a physician, although other profiles have been recognized as physician associates/assistants (42), pharmacists (43, 44), and nurses (45). However, the proportion of CTs with physician associates/assistants as investigators is low (14 in 145.398 in the US 2007–2020) (42), and of pharmacists too (523 in 2009 in the US) (46). Even more, some local regulations may prevent non-physician profiles as pharmacists from being principal investigators (47).

Successful clinical research heavily relies on education and training, as highlighted by various studies (14, 48). However, the relevance of clinical research is typically only a tiny part of basic medical training (40). Clinical specialties provide a significant opportunity for the training of new clinical researchers. Several medical specialty training requirements proposed by the European Union of Medical Specialists include mentions of clinical research with specific reference to CTs (49). Recent recommendations to improve CT investigator training promote using more effective methods of adult learning and focus on real needs, changing the behavior of study personnel and considering the potential challenges associated with a particular protocol, as well as the most common deviations that have occurred in protocols similar in design or therapeutic area (50, 51).

The sponsor usually certifies investigator qualifications before participation in CTs through GCP training (3, 52). Most biopharma companies have developed their own GCP courses, requiring each trialist to attend as a condition for participating in their trials (40). These programs have significant disparities and require substantial time that often does not prepare clinicians for the hands-on investigator responsibilities (53). Moreover, the current content of some GCP training materials is considered redundant, unengaging, and uninteresting (52). Various programs aim to qualify clinical research investigators through mentoring involving multiple activities and clinical departments (14, 54) (Table 4). Despite these recognized drawbacks, investigator certification is associated with improved data quality in clinical research (55). Recommendations to evaluate the impact of GPC training have also been proposed (14).

**TABLE 4:** Examples of clinical research training investigator programs.

Center	Program	Characteristics	Reference
FDA	Clinical Investigator Training Course	Virtual. Two days. Recording available free. Focused on: FDA’s approach to trial design, safety concerns, statistical issues in the analysis, clinical investigator responsibilities	(229)
Mayo Clinic	Clinician Investigator Training Program	Two year. Comprehensive educational experience for residents and fellows interested in pursuing a career that includes research within a robust clinical practice.	(230)
Will Cornell Medicine	CTSC advanced certificate in clinical & translational investigation	One-year program consisting of a multidisciplinary core competency-based curriculum (a total of 10 courses, 22 course credits).	(231)
Clinical Research Training Center. Washington University in St. Louis	Graduate certificate in clinical investigation	16-credit certificate program. For young investigators committed to pursuing academic careers in clinical research	(51)
Pharmatrain federation (22 institutions or associations in Europe)	Specialist in Medicines Development	4-year modular curricula.	(232)

It is worth noting that participating in a trial allows clinicians to be at the forefront of a particular area of medicine. It often enables researchers and their staff to meet with researchers from the same country or worldwide to exchange ideas and plan future collaborations (56).

### 3.3 Principal investigator challenges

Investigators must be aware of their enormous responsibility as their workload significantly increases with more legal, regulatory, financial, and administrative issues (27). CTs demand strict adherence to the protocol, precise and comprehensive documentation of clinical care, and accountability for oversight of all activities related to managing the trial subjects and monitoring the investigational product. Increased trial complexity is derived, at least partly, from new designs that have resulted in significant challenges for investigators, including multiple sample collections, multiple committee approvals, more stringent patient monitoring, complex drug administration, and multiple protocol modifications (57). This has contributed to increased disappointment, less interest in participating in multicenter trials, and a decline in clinicians willing to become clinical investigators (27, 28, 58).

Different barriers for investigators to participate in CTs have been identified as institutional support, infrastructure issues, staff support, administrative burden, time requirements, workload balance, data and safety reporting requirements, and dissatisfaction with finance-related issues (58–61). Accordingly, to these identified barriers, the Clinical Trials Transformation Initiative (CTTI) Strengthening the Investigator Community Project (62) has developed some recommendations focused on reinforcement of four critical categories of site-based research activity: developing site-based research infrastructure and staff, optimizing trial execution and conduct, improving site budget development and contract negotiations, and discovering opportunities for conducting additional trials (58, 63).

The high turnover of CT investigators, believed to lead to inefficiency, instability, and increased costs in conducting CTs, is of concern. A study of the U.S. FDA’s Bioresearch Monitoring Information System showed a decline by approximately one-third of investigators submitting a Form from 1999 to 2015. The proportion of investigators involved in only one trial increased, signaling potential adverse trends in the clinical investigator workforce (64), and yearly, about 40% of unique investigators decide not to participate in another FDA-regulated trial (61).

One of the main operational challenges recognized for CT investigators is recruitment (58, 65). Some studies demonstrate that investigators are optimistic and overconfident in predicting outcomes concerning the statistical significance of their primary outcome and completing recruitment of their registered target enrollment by trial closure (66). Fulfilling patient eligibility criteria and ensuring no exclusion criteria apply before enrolling a patient in a study are two of the most common sources of significant protocol violations (24). The main barriers investigators encounter in recruiting are institutional or clinic time, reimbursement constraints, perceived risk, and treatment preferences (67, 68). Proposals to increase the engagement of investigators other than financial incentives to improve recruitment have been proposed as authorship and letters of accomplishment (66).

Recruitment of under-represented populations in CTs is also a concern, with investigators identifying time constraints and implementation issues as barriers (69). Recommendations for investigators to address this issue have been developed to improve patient communication and provide resources such as transportation or specific apps (69). Published experiences indicate that it may be successfully tackled by offering adequate tools for investigators and monitoring their impact (70). Some studies analyze gender distributions in participants in CTs, detecting a high disbalance with female under-representation (71–73), although this disbalance has improved in the last decade (74, 75). Studies indicate that this distribution is related to gender investigator bias as CT led by women enrolled more female participants (71).

When a physician considers enrolling a patient in a trial, a new relationship develops between the patient and the physician. This relationship could conflict with the traditional doctor-patient relationship. When considering the various treatment arms of a trial (including any placebo arms), the investigator could doubt whether one arm or the other is more effective (76). The “dual-role consent” physician-investigators is particularly relevant to informed consent, especially when they have preexisting treatment relationships, and present recommendations indicate the need to consider the ethical acceptability of dual-role consent that varies with the features of each study. This dual research participation, when approximates usual care, becomes increasingly acceptable—even preferable—for physicians to seek consent for research from their patients (77–79). In pediatric clinical research, the understanding of parents and adolescents is crucial, and the dual role of the physician/investigator presents challenges in communicating the goals of phase 1 trials to families (80).

CTs are changing rapidly due to advancements in technology and new designs. Implementing these novel CT designs, such as basket, platform, and umbrella trials, which have become valuable tools in modern drug development and regulatory processes, adds complexity and new challenges (57, 81). Innovative approaches such as blockchain protocols have been proposed to enhance transparency and traceability of consent, reflecting the evolving landscape of informed consent in clinical research (82). Artificial intelligence tools can combine patient data (demographic, laboratory, imaging, and other -omics) to match patients with those complex inclusion criteria, ensuring recruitment fitness. They may help interpret data and transform it into usable insights to improve patient safety (83). Digital technologies can also improve informed consent and data collection, including patient-reported outcomes, endpoint generation, and compliance monitoring (84, 85).

Digitalizing CTs is undoubtedly an opportunity to improve CTs’ participant access, engagement, trial-related metrics, and intervention efficiency. The use of digital technologies in CTs must consider user attitudes, practices, expectations, and preferences, as potential participants may view using their data (86). Investigators are generally willing to implement new technologies in CT. However, the use of various platforms, interoperability issues, limited technological support, and the potential increased burden caused by them are challenges identified by investigators for its implementation in CTs (87). Still, it also implies the need for new guidance for deploying CTs and new skills in the professionals involved (84, 88).

## 4 Nurses in clinical trials

### 4.1 Clinical trials nurses roles and profiles

Although the need for clinical research nurses (CRNs) has emerged as the vanguard of public awareness during the COVID-19 pandemic, this nursing specialty has supported clinical research for decades (89). An estimated 10,000 nurses are involved in clinical research work in the United States and around 12.000 in the United Kingdom, across a broad spectrum of roles and settings (90). Independent of the position, the demand for CT nurses is increasing (91, 92).

Different names are used to design nurses dedicated to clinical research, some related to specific roles of nurses in CTs, such as CT nurses, study nurses, research nurses, research nurse coordinators, and clinical research nurses (93–96). Concerning the job titles, a variety has been identified among nurses that give support to CTs as a biostatistician, clinical nurse specialist, clinical research associate, coverage analyst, medical data review manager, protocol interpreter, and rural nurse specialist, which reflects a broad spectrum of roles and positions of the nurses (89).

The National Institutes of Health Clinical Center considers CRN a nursing practice focusing on clinical research. It includes care provided to research participants and activities supporting protocol implementation, data collection, and research participant protection. In addition to providing and coordinating clinical care, CRNs have a central role in assuring ongoing maintenance of informed consent, integrity of protocol procedures, accuracy of research data collection, and education of participants (96, 97).

Some reviews classify CRN roles in four areas: participants and managers of CTs, caregivers and protectors of subjects, coordinators of research teams, and educators (96). In an international study, a variety of deployed roles of CRNs have been identified (Table 5), being the ones more uniformly present in the nine countries involved, acting as a central contact for participants and the research team, documenting participant data, monitoring patient safety, educating participants, participate in the informed consent, assessing, and advocating participants in CTs, reporting adverse events, and documenting patient care and process specimens (93). Another recent European study identified that the most frequently assigned duty for CRNs was administering investigational medicinal products, processing blood samples, and shipping clinical samples (96). Concerning time spent by CRNs in CTs, patient care is the main task of CRNs, which includes monitoring patients for adverse events, teaching participants about the study, reporting potential patient adverse events, recording patient research data, or explaining study procedures to patients (98).

**TABLE 5:** Main roles areas for CRNs and their level of deployment the world [Modified of (93)].

CTN roles	CRN involvement (1 to 5 +)
Research teams: central contact, documents, monitor, team education, protocol management	+ + + +
IRB/independent ethics committee and informed consent development: informed consent evaluation, patient education, IRB member	+ + +
Protocol assessment tasks: protocol feasibility, submission, review	+ +
Study participants: informed consent, patient assessment, communication and advocate with participants, scheduling	+ + + + +
Study site management: monitor, documents care, communications, manages and negotiates budget, processing samples	+ + + +

The role of CRNs is particularly relevant in some CT activities that require clinical expertise, dialogue skills, and proximity to the patient, such as in the deployment of informed consent. CRNs can assist in designing, reviewing, and approving informed consent (96, 99). CRN guidance and organizational skills in communication and proximity to the patient are also relevant in educating participants about consent documentation and helping them understand the information on the consent form. This supporting role for informed consent is especially relevant in cases of limited health literacy that conditions a lack of comprehension of CT consent documents, increased anxiety during the informed consent process, and even the success in CT enrollment (100).

The literature supports nurses’ relevance in CTs, as they promote an increase in recruitment (101), enhance subject retention (102), and increase general study efficiency (103). Nurses are also viewed as specifically relevant in deploying retention strategies based on individualizing and identifying threats to CT participants’ retention (102). Some experiences in the UK National Health Services highlight the role of CRNs in building local research, changing research culture, informing the communities, networking, and patient public involvement (104).

Independently of the various roles and responsibilities of the CRNs, the literature highlights that they must continually balance the requirements for protocol integrity and data quality with the clinical needs, comfort, and safety of research participants (105, 106).

In this plethora of roles, two are the central conceptual positions of nurses in clinical research: CRN and research nurse coordinators (95). CRNs, or clinical research care nurses, are clinical research staff nurses with a central focus on the care of research participants. They support study implementation within the context of the care delivery setting. Research nurse coordinators are primarily responsible for study coordination and data management, with particular focus in managing subject recruitment and enrollment, consistency of study implementation, data management and integrity, and compliance with regulatory requirements and reporting (107).

### 4.2 Clinical trial nurses qualification and training

National Institutes of Health have differentiated CRNs’ skills into five categories: care coordination and continuity of clinical practice, human subjects’ protection, study management, and contribution to science (108). These five categories are deployed through 53 different activities, which comprise the full range of practice of clinical nurses providing research-based patient care and study coordinators managing studies (108). Others have proposed an eight-dimensional model of CRNs: care, work-study, expert, lead, prepare, data, advance science, and ethics (98). More recently, an expert team has designed a CRN training model based on the theoretical basis of position competence through literature study, qualitative interviews, and the Delphi method (109).

CRN was recognized as a specialty in the United States by the American Nurses Association in 2016, a relevant step that creates a conceptual basis for developing specialty practice tools such as job descriptions, practice standards, competency assessment, educational content, and certification (110). In this sense, the creation of the International Association of Clinical Research Nurses (IACRN) as a professional nursing organization dedicated to defining, validating, and advancing the specialty practice of clinical research nursing focused on “*maintaining the equilibrium between the care of the research participant and fidelity to the research protocol*” has been a breakthrough. The IACRN supports the professional development of CRNs with specific resources and meetings (110) and has created a curriculum for nursing school adoption and integration of research nursing within accredited programs (111). Oncology CRN has been considered a subspecialty (112), and some specific resources have been developed, such as the Manual for Clinical Trials Nursing (113), a description of their competencies (114, 115), and a CTs Nurse Questionnaire used to determine the various activities of the CRNs within oncology (116).

Australia, where CRN is also a specialty, has developed CRN standards (Table 6). In Europe, a recent survey has identified the presence of specific educational courses for research nurses in the majority of countries. However, the length of the research nurse education and the providers of research nurse courses exhibited significant variation, ranging from workers’ unions to individual institutions or hospitals. Completing the GCP course and renewing the GCP accreditation are regularly mandatory for CRNs in most countries (96).

**TABLE 6:** CRN Australian standards for practice (233).

Applies critical thinking and research knowledge to nursing practice, clinical care, and management of research participants.
Establishes and maintains respectful, diverse, and collaborative professional relationships to promote person-centered research.
Maintains proficiency of practice in both clinical and research disciplines.
Plans nursing care for clinical research participants, applying evidence-based clinical skills and validated and standardized research processes.
Delivers safe, appropriate, responsive, and cohesive care to clinical research participants.
Applies specialized research and management skills to enable the delivery of high-quality clinical and research practice.
Contributes to the advancement of evidence-based health care and the clinical research nurse specialty.

Specific CT training for nurses may positively impact their confidence, ability to discuss CTs, and perceived behavioral norms surrounding such talks. A randomized CT demonstrated that an online video-based educational program increased oncology nurses’ intention to discuss CT with patients and increased self-reported discussions about CT compared to a text-based educational intervention (117).

### 4.3 Clinical trials nurse challenges

Recent data indicate that the number of CRNs in the United States is decreasing (118), which is a matter of concern (119), considering that few nursing schools describe research nursing as a potential career (111). While it’s important for nurse professionals to conduct research (120), CRNs may face difficulties balancing regulatory compliance requirements, research integrity, recruitment targets, and eligibility criteria with their responsibility to prioritize the needs of research subjects and advocate for them. More research is needed to address these challenges (121, 122). Transitioning from nurse to CRN is, per se, a challenge and takes time. It implies thinking like a researcher rather than a clinician, adapting to functioning independently, gain and using skills in data management (123).

The perception of CRNs about their participation in CTs has been analyzed, concluding that most of them remark that they contribute to the clinical research enterprise through knowledge of the research process and nursing expertise in the care of clinical research participants, with an outstanding role in participant care and safety (89). Some studies have analyzed the main barriers for nurses participating in CTs. Lack of knowledge and confidence, concerns about the skills needed to communicate effectively in various patient situations, and the need for explicit norms regarding their role have been identified as relevant barriers (124). Issues like threats to voluntariness, measures to safeguard voluntariness as a time to consider participation, the inclusion of vulnerable groups, and the questionable exclusion of certain groups due to language or cognitive barriers concern CRNs (125).

The decentralization of CTs boosted by the COVID-19 pandemic implies a new model of working for nurses in CTs as home care is incorporated as standard practice. It implies that digital technologies are required, and updating nurses’ competencies is essential in this new CT deployment model, implying an added effort (111).

## 5 Clinical research coordinators

### 5.1 Clinical research coordinator roles

Clinical research coordinators (CRCs) are also known as “study coordinators,” “data managers,” “clinical trial administrators”, and “clinical research associates” (126–128). They are not described or defined in the GCP (129). The Association of Clinical Research Professionals (ACRP) (130) states that a CRC, or Study Site Coordinator, works at a clinical research site under the immediate direction of a principal investigator whose research activities are conducted under Good Clinical Practice regulations.

The tasks developed by the CRCs may be classified into eight domains (Table 7) and are most frequently related to the domain of monitoring activities, to a lesser extent to administrative activities, being researcher-related activities tertiary (126). Among other delegated tasks, CRCs perform site preparation, patient screening and recruitment, patient enrollment, conduct and ensure the quality of case report forms, maintain source documents, and ensure site quality (11, 131–133). CRCs are complementary in providing informed consent to investigators (134, 135). So, although the investigator is responsible for the overall conduct of the CT, the CRCs are responsible for many of the daily activities (136). CRCs may also help in the protocol assessment during the CT conceptualization and evaluation regarding operational logistics or procedures issues (135).

**TABLE 7:** Clinical trial coordinator’s domains of activity and tasks.

**1. Administrative activities**
Management of IRB submission
Management of Hospital Director submission
Management Medicine Agency submission
Medical history adaptation for the CT
Scheduled protocol specified tests
Scheduled patient’s CT appointments
**2. Clinical activities**
To identify potential eligible patients
Assessment of inclusion/exclusion criteria
Participation in informing of patients
Participation in obtaining informed consent
To assess response to therapy
To assess toxicities
Scales/questionnaires completion
**3. Monitoring activities**
Patient registration/randomization
To deal with Hospital Pharmacy
To deal with a central lab
Recruitment follow-up
CRF completion
To act as CRA
To collaborate with the CRA
Queries resolution
Reporting serious adverse events
To handle the investigator file
To prepare and/or attend audits
**4. Data management and statistics**
Database set-up
Data entry
Statistical analysis
**5. Researcher-related activities**
Participation in protocol/CRF design
Participation in protocol/CRF review
Attending investigators meeting Participation in Final Report
Participation in CT publication

Although CRCs usually work in direct contact with healthcare, patients, and patient information, in Western countries, their hiring frequently depends on the investigator and is not linked directly to healthcare systems (137). In Japan, CRCs work under the discretion of their hospital and, in general, support clinical trials in various areas (138).

From the organizational point of view, CRCs may have different levels of responsibilities. Some clinical trial units have established first and second levels of CRCs that are differentiated by the type of studies they manage (minimal risk vs. non-minimal risk CTs) and the kind of tasks (abstracting and screening vs. regulatory and start-up approval). Others have established four skill levels with different experience and salaries (129, 139).

Some critical elements for a good CRC have been considered related to the “five 5 Cs”: coordination, connection, commitment, communication, and collaboration, highlighting the relevance of their responsibilities and networking abilities in CTs (136). It has been valued that CRCs mobilize altruism to deploy their activities, motivate participants to adhere to study protocols, and manage the tension between research and care (140).

Investigators recognize the critical role of the CRCs in the informed consent process, as they have more detailed knowledge of research-related logistics, such as the duration and frequency of visits, possible costs, and compensation, and can offer information about the potential participation requirements. CRCs usually have more time for supporting information, offer a more friendly language, and demonstrate a high degree of that may promote voluntariness (138). CT participants may be more comfortable saying no to coordinators than physician-investigators, which mitigates physician-investigators negative dual role (141). In this sense, CRC’s cultural and racial diversity may facilitate recruitment and CT participant engagement and diminish bias in recruiting marginal populations (142).

CRCs also facilitate adequate billing of CTs. Billing requires information and communication from many sources that must be coordinated, and complex decisions must be documented to avoid common pitfalls covered by the CRC’s role and expertise. Given the potential errors in this process, a CRC can provide the right information punctually to each stakeholder to facilitate an organized and transparent process (143).

CRCs’ responsibilities impact the quality of CTs in a relevant way, and some published audits highlight their relevance (144) and are considered an essential element for an optimal CT unit infrastructure. In an Italian survey, more than 80% of participating sites associated improving the quality of clinical research with implementing a coordinator as a team member (145). CRCs also positively impact scientific output (146).

Different CRC workload assessment tools have also been developed that consider CT complexity to define the optimal number of studies to be assigned to each CRC based on its complexity. These tools consider issues like type of funding (profit or non-profit CTs), frequency of visits, and number of on-site patient access and centralized procedures (147).

### 5.2 Clinical research coordinators qualification and training

The Joint Task Force for Clinical Trial Competency of the Association of Clinical Research Professionals (ACRP) has defined core competencies for Clinical Research Coordinators (148). These competencies are described in a guideline and a certification pathway comprising eight domains. The domains are categorized as entry-level, intermediate, and senior CRCs. The guideline provides self-assessment and competence gap analysis tools for CRCs. The certification pathway includes personalized professional development plans. The guideline and certification pathway is a roadmap for research sites to support CRCs’ hiring, assessment, and development (Table 8). These core competencies are differentiated by the clinical study method (observational vs. interventional) and are associated with potential utilization and outcomes, including training initiatives, job descriptions, and policy development (149).

**TABLE 8:** Group of Domains of competencies of clinical research coordinators developed by the Association of Clinical Research Professionals. Modified from (148).

Scientific Concepts: Encompasses Knowledge of Scientific Concepts Related to the Design and Analysis of CT.
Ethical and Participant Safety Concerns: Encompasses Care of Patients, Aspects of Human Subject Protection, and Safety in the Conduct of a CT.
Investigational Products Development and Regulation: Encompasses Knowledge of How Investigational Products are Developed and Regulated.
Clinical Study Operations (Good Clinical Practice): Encompasses Study Management and GCP Compliance; Safety Management (Adverse Event Identification and Reporting, Post-Market Surveillance, and Pharmacovigilance), and Handling of Investigational Product.
Study and Site Management: Encompasses Content Required at the Site Level to Run a Study (Financial and Personnel Aspects). Includes Site and Study Operations (Not Encompassing Regulatory/GCPs).
Data Management and Informatics: Encompasses How Data are Acquired and Managed During a CT, Including Source Data, Data Entry, Queries, Quality Control, and Correction and the Concept of a Locked Database.
Leadership and Professionalism: Encompasses the Principles and Practice of Leadership and Professionalism in Clinical Research.
Communication and Teamwork: Encompasses All Elements of Communication Within the Site and Between the Site and Sponsor, CRO, and Regulators. Understanding of Teamwork Skills Necessary for Conducting a CT.

The literature has reflected some discussion about the optimal CRC background. Some consider nurses to be the best profile for CRCs with the advantage of having a possible dual role (clinical and CTs support role) that allows them to act as delegates for the investigating physician (including concomitant medications and adverse events (150) while others believe that the general background is considered an advantage (126). Various general skills needed for CRCs are service orientation, negotiation, management of personnel resources, and instructing abilities (133).

Specific CT training of CRCs is based on GCP as the international standard. Although different formal programs are available, surveys to CRCs indicate that these are considered helpful in the initial introduction as a new employee and for refreshing baseline knowledge, although experience, day-to-day practice, observing peers and colleagues, and having mentors are the essential elements to learning (151). Formal training in GCP can improve protocol adherence and clinical trial quality, and one retrospective study data indicates that the number of protocol deviations was significantly lower if the CRCs were certified in GCP (152). Also, educating and training senior CRCs of new teams is considered necessary (2).

Different tools for competency self-assessment have been developed as the various versions of the Competency Index for Clinical Research Professionals, with excellent psychometric properties and a good ability to distinguish between experienced and non-experienced CRCs (153).

### 5.3 Clinical research coordinator challenges

Despite CRCs’ recognized role in CT deployment, some discussions have been opened about their invisible role, and they have sometimes been referred to as “phantom investigators” (145). This is due, at least in part, to the fact that specific certifications infrequently recognize the CRC professional profile (129), and no formal CRC position exists in institutions’ staff, as required by national healthcare workforce regulations (127). For example, in the United States, the Bureau of Labor Statistics does not recognize CRCs (129). However, the demand growth in the market research management of CRCs is objectively relevant (154).

Also, some studies reflect CRC’s inadequate professional identity, suboptimal remuneration, and lack of peer support and recognition (155). CRCs’ job satisfaction and retention have been issues of discussion as turnover is relatively high and impacts productivity and emotional costs. An adequate salary, greater respect, collaboration, and engagement from the principal investigator are significantly associated with higher retention (155, 156). Over-delegation of responsibilities and under-supervision to CRCs is another issue of discussion identified in warning letters of the regulatory agencies (156). In this frame, some have warned about the risk of high turnover, lack of identity of job description, and lack of institutional support (157).

Some proposals for clarifying the competencies for specific clinical research roles have been established by the Special Programme for Research and Training in Tropical Diseases (TDR) of the World Health Organization (158) and by the Multi-Regional CTs Center at Harvard University (13). As the CRC job is not well delineated and competencies are increasingly broad, difficulties have been described in its recruitment, due at least in part to its description in job offers. A guideline for human resources units recruiting CRCs has been elaborated to overcome this barrier (139).

Several challenges for the CRCs in CTs are related to recruitment, specifically in racial and ethnic minorities. Fronting to this population implies overcoming language and cultural barriers and emotional challenges associated with requesting participation from seriously ill patients. Adaptive and technical strategies to improve recruitment of underrepresented CT participants from the CRCs’ point of view have also been identified (159).

CRCs’ burnout is a concern (160). Causes for CRCs’ burnout are diverse and associated with job dissatisfaction, perceived daily work overload, low endurance, and nurturance personality traits. As the burnout risk of CRCs has been identified with high rates accordingly, some studies about some team-based interventions have been developed demonstrating that they are affordable with good results (139, 161).

## 6 Clinical trial pharmacist

### 6.1 Clinical trial pharmacists roles and profiles

CT pharmacists have differentiating roles and expertise that impact the organization and activities of CTs (162). Some specifications about the pharmacist’s responsibilities in CTs are described in the GCP (3), which defines the need for dispensing records kept at the pharmacy and notes that an investigator should delegate responsibility for the storage and accountability of investigational products to an appropriate pharmacist. In the United States, the Code of Federal Regulations defines issues related to investigational product management (163).

Pharmacists CT support is usually provided by an investigational drug research service (clinical research pharmacy) that can be a small-scale operation with a part-time pharmacist or a large-scale operation with a team of dedicated clinical research pharmacists, technicians, and coordinators. Usually, it is a component of a more prominent pharmacy organization, such as a hospital pharmacy (162). Specific investigational drug service pharmacists are present in many centres (164), and the literature highlights that investigational drug service management is especially relevant in supporting early-phase CTs (165). Guidelines (162), best practices standards (165, 166), and frameworks (164) for investigational drug services provided by pharmacists have been published, making medication management a central issue of their tasks.

The Society of Hospital Pharmacists of Australia (166) defines standard roles (Table 9), services (Table 10), and the following objectives for investigational drug research services:

**TABLE 9:** Role of the clinical trials pharmacist accordingly the Society of Hospital Pharmacists of Australia (166).

Coordinating, collaborating, and providing support for the CT pharmacy service
Delivery of pharmacy services that improve participant medication outcomes and add value to health care systems, while encouraging the financial sustainability of healthcare
Development of and input into policies, procedures, guidelines and resources
Commentary on CT protocols
Provision of education and training for healthcare
professionals and students
Provision of education and counselling to clinical
trial participants, carers, medical and nursing staff
and other pharmacists
Pharmacy research related to CT

**TABLE 10:** Minimum and additional services required for a clinical trials pharmacy service [adapted from (166)].

**Minimum pharmacy services**
• Investigational product management including: receipt, storage, temperature control, quarantine, return, authorised destruction, preparation and dispensing of all investigational products. Interactive voice/web response system use.
• Involvement in compounding or manufacturing investigational products
• Provision of emergency 24-h access to the service
• Procedures to ensure compliance with protocols
• Liaising with the investigator(s), trial coordinators, and sponsor representatives
• Provision of information and education to participants and carers, medical and nursing staff and other pharmacists as required
• Monitoring of participant compliance
• Provision of information to, and involvement in the institutional review of protocols, via membership of a scientific review committee and/or Human Research Ethics Committee (HREC)
• Retention and archiving of records
**Additional pharmacy services**
• CT design
• Preparation of blinding plans and unblinding procedures
• Protocol development
• Randomisation codes (e.g. for blinded CT)
• Preparation of placebos and special dosage forms
• Adverse drug reaction reporting
• Literature searches
• Therapeutic drug monitoring
• Advising on regulatory and non-regulatory aspects of conducting CT
• Collection and analysis of data
• Education of pharmacists, pharmacy students, and other healthcare professionals
• Importation of investigational medicine material
• Distribution of investigational products to other study sites
• Management of the financial aspects of the CT

• Provide safe and ethical use of investigational products by ensuring they are appropriate for use and are procured, handled, stored, and used safely and correctly.
• Apply the principles of best pharmacy practice to evaluate new investigational products or medicines.
• Ensure that the pharmacy aspects of investigational product use comply with relevant legislation, standards, guidelines, and local or institutional policies.
• Consider the safety and welfare of participants and the protection of their legal and ethical rights, including confidentiality and privacy.

Differentiating requirements for investigational drug research services according to the complexity of the supported CTs has been proposed. For example, for first-in-human CTs, adaptive trials, and multidrug regimens that have a high risk for adverse events or drug–drug interactions, high standards have been recommended. These imply the needing for an on-call investigational drug service pharmacist to conduct concomitant medication reviews, assess potential drug–drug interactions, monitor renal or hepatic function as a basis for dose adjustment, deliver initial patient education on the investigational drug, and advise patients on any necessary dose modifications (164).

Some of the CT pharmacist’s roles are specific to investigational drug services such as frozen storage, drug accountability, expiration date tracking, manufacturing, compounding, labelling, blinding of investigational drugs, subject and patient training, dispensing and dosing, monitoring drug events, importation and distribution of investigational medicine material, and maintaining written and up-to-date Standard Operating Procedures for the handling of investigational products (164–169). However, other pharmacists’ roles may overlap with other CT support profiles, such as CT design, protocol development, volunteers’ recruitment, adverse drug reaction reporting, data collection and analysis, education, financial management, or IRB participation (170, 171).

In a national survey developed in the United States, 26 pharmacy services supported cancer CTs over 61 centres. Services were classified as pretrial implementation support, trial implementation support, patient medication profile review, medication therapy engagement, medication therapy management, and miscellaneous. The most common pharmacy-performed services (over 60% of respondents) were a clinical review of investigational drug prescribed orders before dispensing, the recommendation for dose adjustments, a review of trial protocol for scientific merit, the development of training material for research staff, and pharmacist IRB membership. The least frequently performed services (under 10% of respondents) were presenting or publishing trial findings, serving as study co-investigator, consenting patients for trial participation, and serving as principal investigator (172).

The potential impact of pharmacists in CTs is relevant in the medication reconciliation process and especially in identifying protocol-prohibited concomitant medications (173). Despite the recognized role of pharmacists in drug interaction screening during eligibility assessment, they are only sometimes involved in this phase. However, pharmacists were more frequently involved in drug interaction screening for enrolled patients with medication changes (174, 175).

During a patient’s CT participation, pharmacists can create a personalized ‘concomitant medication review guide’ in table presentation. This guide can assist other clinicians in preventing and assessing drug-drug interactions by listing critical medication-use information (176). This has been considered of particular relevance in CTs as blind study participants to treatment groups impact product appearance and packaging, lacking differentiating features such as colour or font, being necessary, including a warning about investigational use (168).

The comprehensive medication review, and specifically the resolution of drug–drug interactions, for participants in oncology CTs has been considered crucial as nearly 70% of the patients may have drug interactions (176, 177). The relevance of this role is reinforced by the fact that frequently the CTs protocols do not asses adequately the interactions (178) and that patients enrolling in CTs often require medication changes to meet eligibility requirements (177).

In IRB participation, pharmacists add complementary relevant expertise (162, 179). For example, in the protocol design, a pharmacist may define the parts related to drug information and medication management, including investigational drug management. This would cover the drug information section of the research protocol, as well as the assessment of the informed consent form for appropriate language on benefits and hazards related to adverse drug reactions, drug information supplements, patient logs, or other adherence procedures. In the review of the protocol for the IRB, pharmacists have specific training to evaluate the quality and practicability of the drug supply management, administration, and dosing strategy, the pertinence of additional regulatory requirements (e.g., Risk Evaluation and Mitigation Strategy programs), and medication safety strategies (165, 179, 180).

Pharmacists may also participate in the screening, assessing, and recruiting volunteers in CTs (181–185). Several experiences involving pharmacists in managing patients in clinical research in primary care have been communicated, with studies focused on pharmacological treatment of acute low back pain (181), preventive intervention of cardiovascular events (185), atrial fibrillation screening (183), and assessing outcomes of enhanced chronic disease care through patient education (186).

The benefits of pharmacist participation in CTs have been described. Different surveys indicate that pharmacists consider that their participation in CTs implies improved relationships and communication with other healthcare professionals, enhanced training and knowledge, exploration of personal interests, and development of the pharmacy profession (172, 187, 188).

New models of work using remote communication workflows have implied readaptation of the investigational drug services. Site qualification visits, site initiation visits, sponsor monitoring visits during a clinical trial, mailing of oral investigational products to patients, and virtual sponsor audits have been widely implemented following the coronavirus disease 2019 (COVID-19) pandemic outbreak and are now standards. Its use must be adapted to the characteristics of each CT and patient (163).

### 6.2 Clinical trial pharmacists qualification and training

Specific certifications for clinical trial pharmacists are less abundant than other CT support profiles (189). The American Society of Health-System Pharmacists (ASHSP) has defined five required competency areas, 14 goals, 51 objectives, and 305 criteria for investigational drugs services and research pharmacy residence programs (Table 11). This program defines the 69 key topics designed to provide comprehensive training for the residents across a spectrum of research concepts, regulatory requirements, performance measures, information technology, and leadership principles (190, 191).

**TABLE 11:** Required competency areas and goals defined by the for investigational drugs and research pharmacy residencies (190).

**Competency Area R1. Patient Care**
Goal 1. In collaboration with the health care team, provide comprehensive medication management to research participants following a consistent patient care process.
Goal 2. Ensure continuity of care during research participant transitions between care settings.
Goal 3. Manage and facilitate delivery of investigational products to support safe and effective drug therapy for research participants
**Competency Area R2: Advancing Practice and Improving Patient Care**
Goal 1. Manage the medication-use processes for investigational drug services, as applicable to the organization.
Goal 2. Demonstrate ability to conduct a quality improvement or research project
**Competency Area 3. Research Protocols and Regulations**
Goal 1. Serve as an authoritative resource on the optimal use of investigational products used in clinical research.
Goal 2. Demonstrate ability to assess the feasibility of research protocols for the organization.
Goal 3. Serve as an authoritative source to the IRB) to ensure the safety of human subjects.
Goal 4. Demonstrate the ability to evaluate the federal, state, and institution regulations as it pertains to CT.
**Competency Area R4: Leadership and Management**
Goal 1. Demonstrate leadership skills for successful self-development in the provision of care for research participants.
Goal 2. Demonstrate management skills in the provision of care for research participants.
Goal 3. Demonstrate skills in the financial management and budgeting to conduct CT.
**Competency Area R5: Teaching, Education, and Dissemination of Knowledge**
Goal 1: Provide effective CT and medication education to participants, caregivers, health care professionals, students, and the public (individuals and groups).
Goal 2. Effectively employ appropriate preceptor roles when engaged in teaching students, pharmacy technicians, or fellow health care professionals about care of research participants and investigational drug services.

Accordingly, several programs focused on Investigational Drugs & Research pharmacy residency training have been developed, some accredited by reference associations such as the ASHSP. The training program requires at least 3,000 h of fellowship devoted to research-related activities over 2 years. Detailed information about the research pharmacy residency in reference hospitals is accessible (192, 193).

### 6.3 Clinical trial pharmacists challenges

Implementing investigational drug services and high-quality drug management in CTs implies a comprehensive approach. It needs to provide adequate resources and implement detailed processes that involve computer software, barcoding, expiration date tracking, order templates, clinical decision support, and pharmacy-independent double checks at the point of dispensing, in addition to many of the legal and accreditation requirements already mentioned (165).

One of the main concerns for CT pharmacists is medication safety. Safe practices in medication management in CTs have not been standardized, and several areas of the operative medication-use process may pose specific safety risks during clinical research. For example, the repackaging process implies contamination risks. Storing investigational drugs may require particular facilities as they must be separated by protocol and strength to reduce the risk of incorrect product selection during dispensing (165, 168).

Perceptions toward practice-based research of pharmacists have also been analyzed in the literature, showing a consistently high degree of interest in research in general, with percentages of 70–87%. However, studies show high variability in the proportion of pharmacists who had experience with practice-based research (30–77%) and were confident they could conduct practice-based research (34–73%) (172, 188). Different surveys indicate that the main barriers identified are low research training and management priority, lack of funding, time, resources, and culture, as well as opportunities and awareness of opportunities (172, 187, 188).

Although not specific to CTs, deploying computerized provider order entry systems is complex. It implies a straight collaboration framework with clinicians to contribute to the safer use of medications (194). Integrating electronic health records in CTs can increase generalizability, reduce costs and time, expand the research fields, and associate with various stakeholder benefits (195). However, it poses significant challenges, including infrastructure costs, interoperability, standardization, data quality, ethics, privacy, and data security considerations. The implementation involves different stakeholders in the healthcare arena, CT pharmacists being one essential (196).

3D printing helps facilitate rapid CT prototyping, automate compounding processes in pharmacies, and ensure precise dosages and drug combinations while enhancing precision, efficiency, and patient safety (197, 198). However, there are still obstacles, such as regulatory considerations, material selection, and quality control. Despite these challenges, 3D printing is a promising technology that can revolutionize pharmaceutical innovation due to ongoing advancements.

## 7 Clinical trial monitor

### 7.1 Clinical trial monitors profile and roles

The CT monitor (or clinical research associate) is responsible for overseeing the progress of a CT and ensuring that it is conducted, recorded, and reported according to the protocol, standard operation procedures, GCP (3), and the Medicines for Human Use Regulations (199). GCP (3) explicitly states that the sponsor should appoint monitors according to the extent and nature of monitoring based on considerations such as the objective, purpose, design, complexity, blinding, size, and endpoints of CTs.

Information about monitoring principles and different guidance are available on the websites of the leading international regulatory agencies such as the European Medicines Agency, FDA, Medicines and Healthcare Products Regulatory Agency, or other research or research supporting institutions such as CTs Transformation Initiative, Health Research Authority, International Council for Harmonisation of Technical Requirements for Pharmaceuticals for Human Use, National Institute for Health Research, TransCelerate Biopharma Inc or UK Trial Manager Network (199).

Five main purposes have been defined for CT monitors: that the rights and well-being of the human subjects are protected, the reported trial data are accurate, complete, and verifiable from source documents, the conduct of the trial is following the currently approved protocol/amendment(s), GCP and the applicable regulatory requirements, improve the way the trial is running and prevention of pitfalls (3, 199, 200). According to GCP (3), these five aims are deployed in a list of responsibilities (Table 12).

**TABLE 12:** Monitor responsibilities [modified from (3)].

Acting as the main line of communication between the sponsor and the investigator.
Verifying that the investigator has adequate qualifications and resources
Verifying, adequacy of the storage, supply and return, information given respect the investigational product(s)
Verifying that written informed consent was obtained before each subject’s participation in the trial.
Ensuring that the investigator receives the current Investigator’s Brochure, all documents, and all trial supplies needed to conduct the trial properly and to comply with the applicable regulatory requirement(s), and that the investigator and the investigator’s trial staff are adequately informed about the trial.
Verifying that the investigator’s trial staff are performing the specified trial functions, enrolling only eligible subjects, and providing all the required reports, notifications, applications, and submissions, and that these documents are accurate, complete, timely, legible, dated, and identify the trial.
Reporting the subject recruitment rate.
Verifying that source documents are accurate and complete, and kept up-to-date and maintained and the accuracy and completeness of the CRF entries, source documents and other trial-related records against each other.
Informing the investigator of any CRF entry error, omission, or illegibility.
Determining whether all adverse events are appropriately reported.
Determining whether the investigator is maintaining the essential documents.
Communicating deviations from the protocol, to the investigator and taking appropriate action designed to prevent recurrence of the detected deviations.

In daily life, monitoring activity requires deploying all the skills related to the different roles they are involved in, such as trainers, planners, trouble-shooters, negotiators, detectives, or psychologists (201). Some of the specific tasks of the monitor during the initial CT visit include determining whether the administrative file is complete and in order, checking that the protocol is signed, and attaching the investigator’s curriculum vitae. After this initial step, the monitor ensures the application and respect of good clinical practices, particularly strict adherence to the protocol, reviews the trial data after having consulted the clinical files, checks their authenticity and coherence, determines that the randomization order has been respected, assures the providing of the necessary products to the investigator for conducting the trial such as drug packages and materials for biological assays, and ensures that communication is maintained throughout the trial (200, 202–204).

Each described CT monitor responsibility is deployed according to standard operating procedures that itemize every task that must be deployed. For example, pharmacy monitoring includes documentation and delegation log review, protocol and SOP compliance, investigational medicinal product storage check, labelling expiry dates randomization, accountability, and breaking blind procedure, and each of these elements includes detailed activities (200, 205).

Planning is a relevant issue related to monitoring activities. A monitoring plan is a relevant part of the activity to ensure optimal monitoring of the CT, according to the specific risks to human subject protection and data integrity of each CT. The plan should describe the monitoring strategy, the responsibilities of all parties involved, the different monitoring methods, and the rationale for their use. The plan should also emphasize monitoring critical data and processes (3).

Although the monitors are external to the organizations, they must have direct access to the original medical records of the trial subjects to verify the trial procedures and/or data (199). This must be done without violating the subject’s confidentiality, to the extent permitted by applicable laws and regulations, and the subject must authorize such access by signing a written informed consent form (3).

The cost of monitoring is relevant. It has been estimated that the average cost per site visit of a monitor in an oncology CT is around 1.500$ (206), and about 25% of the cost of a CT is occasioned by on-site monitoring activities (207). Monitoring activity before, during, and after the CTs is usually developed on-site. However, central monitoring is also an option for specific circumstances considering each CT’s objective, purpose, design, complexity, blinding, size, and endpoints (3).

### 7.2 Clinical trial monitor qualification and training

The Association of Clinical Research Professionals (ACRP) has defined a Core Competency Framework for Clinical Study Monitoring with four different levels (entry-level, intermediate, senior, and clinical lead), which include domain, competency, and expectation (Table 13). More deeply considered issues are related to managing confidential information, the informed consent process, and the need to communicate with the team’s participants in the CTs (130).

**TABLE 13:** Core competency framework for clinical study monitoring (130).

Ethical and Participant Safety Considerations
Medicines Development and Regulation
CT Operations (GCPs)
Study and Site Management
Data Management and Informatics
Leadership and Professionalism
Communication and Teamwork

Indeed, becoming a clinical trial monitor does not necessarily require formal education, as various pathways are available for anyone with a high school diploma or higher (208). Information concerning the academic background of the monitors is limited. In a Children’s Oncology Group report from the National Cancer Institute (209) that analyzed the cause of turnover in a survey of over 456 monitors, the level of education was a bachelor’s degree at 47% and a graduate degree at 28% (210).

Despite the absence of a certification requirement, obtaining certification in the United States can offer more job prospects and even higher salaries (210). Various training programs for clinical trial monitor training are available (211, 212). The Society of Clinical Research Associates (SOCRA) (213, 214), the Association of Clinical Research Professionals (ACRP), Certified Clinical Research Professionals Society (CCRPS) (215), among others, provide certifications. Also, different University programs are available (208).

Updated statistics from the United States indicate that the average salary of a clinical trial monitor is $73,700/year (216). A senior clinical trial monitor (minimum 2 years of clinical monitoring experience, remote work) is around $ 120.000–135.000 per year, and some employment offers include a benefits package and an annual performance bonus. A lead clinical trial monitor reaches salaries (accordingly published offers in the United States) of around $150.000–170.000/year (217).

### 7.3 Clinical trial monitor challenges

There is a growing concern about the effectiveness and efficiency of monitoring practices and a need for empirical evidence to determine which practices best achieve the goals of trial monitoring. Risk-based monitoring has been proposed as an efficient way to cover most of the activities developed by on-site monitoring or even detect flaws better and sooner than on-site monitoring (177, 178, 218, 219). Risk-based monitoring combines centralized follow-up for most of the reviews and on-site for further support and has been studied with results that suggest that it improves data quality regarding data points of major importance to trial outcomes, efficacy, and significant safety requiring less than 50% of extensive on-site monitoring resources (204, 219–222). Based on this data, international recommendations promote risk-based monitoring (3, 223, 224), although most small single-site academic studies apply traditional approaches (91, 221, 225).

CT monitor turnovers are high, and data from different countries indicate that the percentage of annual turnover in 2015–2019 was around 25% due at least in part to a low salary, unmanageable workload, lack of career advancement, and limited research commitment from the medical team (226). The main factors considered to monitor for staying in their jobs are a supportive principal investigator and satisfaction working with colleagues (209).

The CT monitor position and adaptation capability are recognized as valuable. On the other hand, CT monitor are motivated and excited about how they can contribute to the health and well-being of others and their career by rapidly learning critical skills and fast adaptation capabilities. During the COVID-19 pandemic, a rapid transition to decentralizing to monitoring activity has occurred (227). In this scenario, companies are creating retention programs focused on building more connections among professionals and reducing “travel fatigue”, creating a professional pathway and annual incentives (226).

While CT monitors currently play a crucial role in healthcare, their importance is projected to diminish in centralized models where patients can provide much of the necessary information directly and through electronic health records in an automated way. Wearables have immense potential for automated patient monitoring and will be an integral part of the future of healthcare models alongside CT monitoring (228).

## 8 Conclusion

CTs are becoming increasingly complex and require trained teams of expert professionals to support various activities with maximum rigor, adhering to ethical and technical international requirements. Healthcare professionals, including principal investigators, CRCs, nurses, pharmacists, and monitors, have pivotal roles in the successful execution of CT. Clearly defining roles, providing adaptive and continuous professional training, and fostering a culture of collaboration and communication are pivotal in ensuring success in this field. Addressing recruitment, retention, and professional growth challenges is also essential.

Although professional profiles and roles of the experts involved in CTs are not well delineated, some roles and functions are recognized internationally as essential for its deployment, especially in highly complex CTs. In this sense, a relevant advance has taken place in recent years. As CTs gain weight as a relevant part of the activity of worldwide leader healthcare systems, better delineation of roles, training, and a higher degree of professionalization are desirable.

Across all roles, there is a clear need for more standardized, formalized, and accessible training programs to ensure that professionals are equipped to meet the demands of their roles in clinical trials. Also, a collaborative approach and enhanced training programs tailored to each professional role’s specific needs and challenges within the CT framework are convenient. All professionals involved in clinical trials must navigate a complex landscape of ethical and regulatory requirements and new technology advances, underscoring the need for continuous education and adherence to ethical standards.

Specific strategies are needed to front the challenges related to the professionals involved in CT in the healthcare arena. Principal investigators should focus on enhancing protocol compliance through rigorous training in GCP and regulatory updates, ensuring they are well-versed in the latest standards and delegated in adequately trained teams. For clinical research coordinators, implementing project management tools and comprehensive training in patient interaction and data management systems is advised to effectively manage the trial’s increased complexity. Monitors could benefit from advanced training in risk-based monitoring techniques and digital monitoring tools to enhance the efficiency and effectiveness of site visits and remote monitoring tasks. Nurses, who often face direct patient care challenges, should receive ongoing support in patient management systems and be given access to continuous education in trial-specific procedures and treatments. These steps will enhance individual performance and improve clinical trials’ overall functioning, ensuring data integrity, participant safety, and regulatory compliance.

Digitalization can streamline many aspects of CT processes, from patient recruitment and data collection to protocol compliance and real-time monitoring. This improves efficiency, enhances data integrity, and reduces the administrative burden on staff. For instance, electronic health records and mobile health applications could be more extensively utilized to facilitate seamless communication and accurate data tracking.

Future studies could explore the specific impacts of digital technologies on trial efficiency and participant safety, the effectiveness of new training modalities, and the long-term benefits of these innovations on clinical trial outcomes. Research into the development of a standardized framework for integrating digital tools across various stages of clinical trials could also provide valuable insights and guidance for the field.

## Author contributions

GP: Writing–review and editing, Writing–original draft, Visualization, Validation, Supervision, Software, Resources, Project administration, Methodology, Investigation, Funding acquisition, Formal analysis, Conceptualization. BS-S: Writing–review and editing, Writing–original draft, Visualization, Validation, Supervision, Software, Resources, Project administration, Methodology, Investigation, Funding acquisition, Formal analysis, Conceptualization.
